# Prognostic value of quality of life score in disease-free survivors of surgically-treated lung cancer

**DOI:** 10.1186/s12885-016-2504-x

**Published:** 2016-07-20

**Authors:** Young Ho Yun, Young Ae Kim, Jin Ah Sim, Ae Sun Shin, Yoon Jung Chang, Jongmog Lee, Moon Soo Kim, Young Mog Shim, Jae lll Zo

**Affiliations:** Department of Biomedical Science, Seoul National University College of Medicine and Hospital, 103 Daehak-ro, Jongno-gu, Seoul, 110-799 Korea; Cancer Research Institute, Seoul National University College of Medicine, Seoul, Korea; National Cancer Control Institute, National Cancer Center, Goyang, Korea; Department of Preventive Medicine, Seoul National University College of Medicine, Seoul, Korea; Center for Lung Cancer, National Cancer Center, Goyang, Korea; Lung and Esophageal Cancer Center, Samsung Comprehensive Cancer Center, Samsung Medical Center, Seoul, Korea

## Abstract

**Background:**

We aimed to evaluate the prognostic value of quality of life (QOL) for predicting survival among disease-free survivors of surgically-treated lung cancer after the completion of cancer treatment.

**Methods:**

We administered the European Organization for Research and Treatment of Cancer Quality of Life Questionnaire Core 30 (EORTC QLQ-C30), the Quality of Life Questionnaire Lung Cancer Module (QLQ-LC13), Hospital Anxiety and Depression Scale (HADS), and Posttraumatic Growth Inventory (PTGI) to 809 survivors who were surgically-treated for lung cancer at two hospitals from 2001 through 2006. We gathered mortality data by linkage to the National Statistical Office through December 2011. We used Cox proportional hazard models to compute adjusted hazard ratios (aHRs) and 95 % confidence intervals (CIs) to estimate the relationship between QOL and survival.

**Results:**

Analyses of QOL items adjusted for age, sex, stage, body mass index, and physical activity showed that scores for poor physical functioning, dyspnea, anorexia, diarrhea, cough, personal strength, anxiety, and depression were associated with poor survival. With adjustment for the independent indicators of survival, final multiple proportional hazard regression analyses of QOL show that physical functioning (aHR, 2.39; 95 % CI, 1.13–5.07), dyspnea (aHR, 1.56; 95 % CI, 1.01–2.40), personal strength (aHR, 2.36; 95 % CI, 1.31–4.27), and anxiety (aHR, 2.13; 95 % CI, 1.38–3.30) retained their independent prognostic power of survival.

**Conclusion:**

This study suggests that patient-reported QOL outcomes in disease-free survivors of surgically-treated lung cancer after the completion of active treatment has independent prognostic value for long-term survival.

## Background

Health Related Quality of life (HRQOL) is an important clinical outcome for treatment comparisons in cancer patients [1, 2]. Although advances in early detection and treatment strategies have increased the likelihood of survival, lung cancer survivors are known to suffer substantial symptom burdens [3]. Although earlier studies suggested that physical symptoms such as anorexia [4], pain [4–7], and fatigue [4] are the strongest independent prognostic factors for survival even after the adjustment for established prognostic variables, mental health criteria, such as psychological distress, existential well-being, and posttraumatic growth, would also be independent predictive contributors for long-term survival among long-term cancer survivors [1, 8, 9].

QOL is a critical independent prognostic factor for predicting survival [10–12]. However, studies regarding the prognostic value of QOL have primarily focused on QOL at the time of the diagnosis or treatment at baseline [13–19]. Prior analyses have also shown that QOL is an important prognostic factor in patients with advanced lung cancer [12, 20–24]. measured QOL at the time of the diagnosis or clinical treatment trials, and no published studies have focused on the predictive value of QOL for long-term survival in disease-free lung cancer survivors after the completion of cancer treatment. The identification of prognostic factors might help clinicians to correctly survey individuals at highest risk for recurrence and mortality, and allow for appropriate interventions to improve QOL and survival in disease-free lung cancer survivors after the completion of active cancer treatment [11].

Seven years ago, we conducted a large cross-sectional study of QOL among cancer survivors who had undergone primary curative surgery for stage 0-III lung cancer between 2001 and 2006, and were disease-free after primary treatment for lung cancer ended and survived for longer than 1 year without any evidence of cancer [25]. The QOL measurements that we collected allowed us to assess the prognostic value of comprehensive QOL variables, including physical, mental, social, and existential domains, for predicting long-term survival more than 5 years after the survey completion. In this study, we aimed to evaluate the prognostic value of QOL, which provides information on the likely course of cancer mortality by predicting survival among patients with lung cancer after cancer treatment completion.

## Methods

### Participants

In 2007, we conducted a survey of lung cancer survivors. Among 1,633 patients who were contacted at two hospitals in South Korea from 2001 through 2006, we identified 830 survivors who had been surgically-treated for lung cancer. Among them, we excluded 27 subjects whose survival status was censored until December 31, 2011. Thus, a total of 809 patients had been included in this study. All participants provided written informed consent. We collected information regarding the date of the diagnosis, stage, type of treatment, and other clinical characteristics from the hospital cancer registries. This study was approved by the Institutional Review Boards (IRB) of National Cancer Center and Samsung Medical Center.

We collected QOL data among cancer survivors who were disease-free after primary treatment for lung cancer ended and survived without any evidences of cancer for longer than one year. Participants filled out a questionnaire including important survivorship issues such as QOL, anxiety, depression, and posttraumatic growth, etc. This study was approved by the Institutional Review Boards of each hospital. Criteria for enrollment in this study have been previously described in detail [25].

#### Measures

##### Socio-demographic and clinical variables

Through our systematically organized questionnaire, socio-demographic variables (age, sex, level of education, monthly income, employment status, marriage status, physical activity, smoking, and alcohol consumption) were assessed. We considered physical activity (PA) to be at least 30 min of moderate-to-vigorous physical activity for 5 or more days per week (ie, ≥12.5 metabolic equivalent tasks [26]/week). In addition, clinico-pathological data (years from the survey date to the diagnosis date, type of treatment, overweight (a BMI >23 at the time of survey), PA, comorbidities, cancer stage, time since the diagnosis, and the years from survey date to the diagnosis date) were collected from the patients’ medical charts and hospital-based cancer registries.

To determine the influence of comorbidities on cancer patients, we asked patients about the current existence of comorbidities, such as cerebrovascular disease (eg, stroke or cerebral hemorrhage), heart disease (eg, angina pectoris, myocardial infarction, or chronic heart failure), diabetes, liver disease (eg, chronic hepatitis or cirrhosis), pulmonary disease (eg, chronic bronchitis or asthma), hypertension, infectious diseases (eg, tuberculosis, etc.), digestive diseases (eg, chronic gastritis, gastric ulcer, or duodenal ulcer), musculoskeletal disorders (eg, degenerative or rheumatoid arthritis), kidney disease (eg, chronic renal failure, etc.).

### Health-related quality of life (HRQOL)

Patients completed questionnaires that covered the following characteristics: the European Organization for Research and Treatment of Cancer Quality of Life Questionnaire Core-30 item (EORTC QLQ-C30) and lung cancer module (QLQ-LC13), Hospital Anxiety and Depression Scale (HADS), and Posttraumatic Growth Inventory (PTGI).

The EORTC QLQ-C30 is a 30-item cancer-specific questionnaire for measuring global health and overall QOL scales, five functioning domains (physical, role, cognitive, emotional, and social), three symptom scales (fatigue, pain, and nausea and vomiting), and six single items that assess additional symptoms commonly reported by cancer patients (dyspnea, appetite loss, sleep disturbance, constipation, and diarrhea) along with any perceived financial difficulties [18]. The QLQ-LC13 incorporates one multi-item scale (dyspnea) and nine single items (pain in the arm/shoulder, chest, and other organs; cough; hemoptysis; dysphagia; peripheral neuropathy; alopecia; mouth sores). In both surveys, high scores represent better functioning and severe symptoms. HADS is a self-reported assessment tool comprised of two domains: the anxiety subscale and the depression subscale [27]. Each of the two HADS-subscales was measured using seven items rated on a 4-point Likert scale ranging from no feelings of anxiety or depression (0) to severe feelings of anxiety or depression [3]. Total scores ranged from 0 to 21 for each anxiety and depression subscale.

The PTGI includes 21 items regarding positive changes, with five domains relating to others, personal strength, new possibilities, appreciation of life, and spiritual change. Each question was scored from 0 to 5 using a 6-point Likert scale. A higher score signifies greater posttraumatic positive growth [28].

### Survival data

The patients were followed regularly by each hospital registries after the completion of treatment. If the patients died during that follow-up, the family caregivers were asked the date of death. We also gathered mortality data by linkage to the National Statistical Office. We measured survival time from the date of the diagnosis and used mortality data with vital status. The person-years at risk data were accumulated for each patient from the date of the survey to the date of death. During the follow-up of 4509.2 person-years, we identified 96 deaths (11.9 %) among the 809 subjects. In the 809 lung cancer survivors for whom there were available data, the median time from the diagnosis to survey date was 6.0 (±1.24) years and the median survival time was 8.3 (±2.01) years.

### Statistical analyses

First, we performed univariate analyses of the aforementioned demographic and clinical characteristics with the mortality of the lung cancer survivors. Variables that were significant in the univariate analyses were included in the adjusted multiple proportional hazard regression analyses to identify independent prognostic indicators of survival, which formed the baseline prognostic model using a backward feature selection method.

Next, we performed analyses to determine whether HRQOL scores (EORTC QLQ, HADS, and PTGI) were significantly associated with survival using Cox proportional hazard models. Due to the high statistical collinearity problem among the HRQOL variables, each factor was first analyzed separately in the Cox proportional hazard model, which incorporated the baseline prognostic model (specifically age, sex, stage, BMI, and PA) to identify independent HRQOL predictors of long-term survival.

To maximize differences in prognostic strength of QOL scores, we dichotomized each variable score and chose a cut-off point. We dichotomized each scale of EORTC QLQ-C30 and EORTC QLQ-LC13 based on the score for the problematic group: ≤33 on a scale of 0–100 for global QOL or functioning scale, and >66 for symptom scale [29]. Earlier studies with cancer survivors have shown that the scores for the problematic group were useful in identifying the problems of QOL compared with general population [30, 31] In addition, we used HADS as the outcome measure, which was dichotomized with the cut-off point of 8 as a borderline case of anxiety or depression [32]. For PTGI, we dichotomized each variable according to the standardized manual [28].

Finally, we constructed the final model for long-term survival using demographic and clinical characteristics and QOL scores that were identified as independent prognostic indicators of survival with adjusted multiple proportional hazard regression analyses; then, we traced survival curves of the significant QOL factors using PROC LIFETEST. We calculated adjusted hazard ratios (aHRs) and 95 % confidence intervals (CIs). A p-value of less than 0.05 was considered to indicate statistical significance and used to identify significant factors retaining in the model. The SAS statistical package version 9.3 (SAS Institute Inc., Cary, NC) was used for all analyses.

## Results

### Univariate analyses and multiple proportional hazard regression analyses of demographic and clinical characteristics

Table 1 summarizes the baseline demographic and clinical characteristics, as well as crude and adjusted hazard ratios (HRs) for the overall survival from Cox proportional hazards regression models. Table 2 summarized multiple proportional hazard regression analyses using a backward feature selection method with variables that were significant in univariate analyses showed that age, sex, stage of cancer, monthly Income, BMI of overweight indicator, and PA had independent prognostic value.

**Table 1:** Clinical and Socio-demographic characteristics and mortality of lung cancer survivors (*n* = 809)

Variable		No. of deaths/No. of participants	Crude HR	95 % CI		aHR^a^	95 % CI	
Age (years)	<65	33/426	1.00			1.00		
	≥65	63/383	2.28	1.49	3.47	1.99	1.30	3.05
Sex	Female	10/187	1.00			1.00		
	Male	86/622	2.71	1.41	5.22	2.34	1.21	4.53
Education	≥High school degree	20/205	1.00					
	<High school degree	76/604	1.31	0.80	2.15	NS		
Monthly Income(USD)	≥3,000	13/220	1.00					
	<3,000	83/589	2.49	1.39	4.47	NS		
Employment status	yes	30/315	1.00					
	no	66/494	1.46	0.95	2.25	NS		
Currently married	yes	89/744	1.00					
	no	7/65	0.90	0.42	1.94	NS		
Stage	stage 0–I	46/510	1.00			1.00		
	stage II–III	50/299	1.92	1.29	2.86	1.80	1.20	2.70
Comorbidity	no	45/363	1.00					
	yes	51/443	0.93	0.62	1.39	NS		
BMI at the time of survey (kg/m2)	≥23	39/452	1.00			1.00		
	<23	56/355	1.92	1.28	2.89	1.70	1.12	2.57
Alcohol Now	NO	75/623	1.00					
	Yes	21/186	0.92	0.57	1.50	NS		
Current smoking status	no	89/750	1.00					
	yes	7/59	0.99	0.46	2.14	NS		
MET	≥12.5	43/443	1.00			1.00		
	<12.5	53/363	1.55	1.03	2.31	1.54	1.02	2.31
Type of treatment	Surgery	54/485	1.00					
	Surgery and other^b^	42/318	1.47	0.77	2.81	NS		
Years from survey date to diagnosis date	≤3 years	37/343	1.00					
	>3 years	59/466	1.20	0.79	1.80	NS		

*Abbreviations*: *HR* hazard ratio, *aHR* adjusted hazard ratio, *BMI* body mass index, *MET* metabolic equivalent task

^a^Multiple proportional hazard regression analysis adjusted with age, sex, education, income, employment status, stage, comorbidity, BMI, alcohol drinking, current smoking, MET, treatment type, years from survey date to diagnosis date

^b^other was Radiotherapy and Chemotherapy

**Table 2:** Univariate analyses and adjusted proportional hazard regression analyses of QOL

Variable			No. of deaths/No. of participants	Crude HR	95 % CI		aHR^a^	95 % CI	
EORTC-QCQ -C30	Physical functioning	>33.33	87/783	1			1		
		≤33.33	9/26	3.71	1.87	7.36	3.44	1.72	6.88
	Role functioning	>33.33	89/782	1			1		
		≤33.33	7/27	2.51	1.16	5.41	2.14	0.99	4.65
	Emotional functioning	>33.33	92/792	1			1		
		≤33.33	4/17	2.12	0.78	5.77	2.59	0.94	7.14
	Cognitive functioning	>33.33	94/793	1			1		
		≤33.33	2/16	1.08	0.27	4.37	0.95	0.23	3.90
	Social functioning	>33.33	91/786	1			1		
		≤33.33	5/23	1.99	0.81	4.88	1.56	0.63	3.88
	General health status	>33.33	89/770	1			1		
		≤33.33	7/39	1.62	0.75	3.49	1.35	0.62	2.93
	Fatigue	<66.66	83/720	1			1		
		≥66.66	13/89	1.30	0.72	2.33	1.24	0.69	2.24
	Nausea & vomiting	<66.66	94/790	1			1		
		≥66.66	2/19	0.91	0.23	3.73	0.82	0.20	3.33
	Pain	<66.66	87/752	1			1		
		≥66.66	9/57	1.41	0.71	2.80	1.28	0.61	2.66
	Dyspnea	<66.66	53/591	1			1		
		≥66.66	43/218	2.33	1.56	3.48	1.96	1.30	2.95
	Insomnia	<66.66	79/689	1			1		
		≥66.66	17/120	1.24	0.73	2.09	1.27	0.75	2.16
	Appetite loss	<66.66	76/717	1			1		
		≥66.66	20/92	2.20	1.35	3.61	1.68	1.00	2.82
	Constipation	<66.66	87/746	1			1		
		≥66.66	9/63	1.24	0.63	2.47	1.13	0.57	2.27
	Diarrhea	<66.66	88/773	1			1		
		≥66.66	8/36	2.13	1.03	4.39	2.11	1.01	4.40
	Financial difficulties	<66.66	77/686	1			1		
		≥66.66	19/123	1.39	0.84	2.30	1.14	0.68	1.89
EORTC-QCQ -LC13	Dyspnea	<66.66	77/733	1			1		
		≥66.66	19/76	2.62	1.59	4.33	2.26	1.36	3.76
	Coughing	<66.66	77/726	1			1		
		≥66.66	19/83	2.35	1.42	3.88	1.92	1.15	3.20
	Hemoptysis	<66.66	95/803	1			1		
		≥66.66	1/6	1.35	0.19	9.67	0.82	0.11	5.94
	Sore mouth	<66.66	90/782	1			1		
		≥66.66	6/26	2.15	0.94	4.91	1.83	0.80	4.19
	Dysphagia	<66.66	90/783	1			1		
		≥66.66	6/26	2.18	0.96	4.99	1.62	0.70	3.74
	Peripheral neuropathy	<66.66	84/725	1			1		
		≥66.66	12/84	1.28	0.70	2.35	1.29	0.70	2.37
	Alopecia	<66.66	89/756	1			1		
		≥66.66	7/52	1.17	0.54	2.53	1.26	0.58	2.74
	Pain in chest	<66.66	80/727	1			1		
		≥66.66	16/82	1.85	1.08	3.16	1.70	0.98	2.95
	Pain in arm or shoulder	<66.66	82/699	1			1		
		≥66.66	14/110	1.09	0.62	1.93	1.22	0.69	2.15
	Pain in other body parts	<66.66	83/726	1			1		
		≥66.66	12/82	1.32	0.72	2.43	1.44	0.78	2.65

*Abbreviations*: *HR* hazard ratio, *aHR* adjusted hazard ratio, *CI* confidence interval

^a^Each of the multiple proportional hazard regression analysis was adjusted for age, sex, stage, BMI at the time of survey, and MET

### Univariate analyses and adjusted proportional hazard regression analyses of QOL, PTGI, and HADS

Table 2 summarizes the multiple proportional hazard regression analyses using a backward feature selection method with variables that were significant in the univariate analyses showing that age, sex, stage of cancer, monthly income, BMI of overweight indicator, and PA showed that the scores of physical functioning (aHR 3.44, 95 % CI 1.72–6.88), dyspnea (aHR 1.96, 95 % CI 1.30–2.95), anorexia (aHR 1.68, 95 % CI 1.00–2.82), diarrhea (aHR 2.11, 95 % CI 1.01–4.40), and cough (aHR 1.92, 95 % CI 1.15–3.20) for the problematic group were associated with poor survival. Additionally, Table 3 summarizes the crude and adjusted HRs for the association between PTGI and HADS with the risk of overall survival. Survivors with poor personal strength (aHR 2.43, 95 % CI 1.35–4.38), anxiety (aHR 2.55, 95 % CI 1.68–3.87), or depression (aHR 1.73, 95 % CI 1.16–2.60) showed significantly diminished length of survival.

**Table 3:** Univariate analyses and adjusted proportional hazard regression analyses of PTGI and HADS

Variable		No. of deaths/No. of participants	Crude HR	95 % CI		aHR^a^	95 % CI	
PTGI								
Relation to others	≥23	35/332	1			1		
	<23	61/477	1.24	0.82	1.88	1.26	0.83	1.92
New possibilities	≥18	13/177	1			1		
	<18	77/578	1.88	1.04	3.38	1.73	0.94	3.18
Personal strength	≥15	14/237	1			1		
	<15	82/572	2.56	1.45	4.5	2.43	1.35	4.38
Spiritual change	≥5	40/407	1			1		
	<5	56/402	1.45	0.97	2.18	1.34	0.89	2.02
Appreciation of life	≥11	33/361	1			1		
	<11	63/448	1.59	1.04	2.42	1.47	0.96	2.25
Total	≥71	23/279	1			1		
	<71	73/526	1.75	1.1	2.8	1.67	1.03	2.69
HADS								
Anxiety	<8	59/634	1			1		
	≥8	36/170	2.41	1.6	3.65	2.55	1.68	3.87
Depression	<8	45/490	1			1		
	≥8	51/313	1.83	1.23	2.74	1.73	1.16	2.6

*Abbreviations*: *HR* hazard ratio, *aHR* adjusted hazard ratio, *CI* confidence interval, *PTGI* posttraumatic growth inventory, *HADS* hospital anxiety and depression scale

^a^Multiple proportional hazard regression analysis adjusted with age, sex, education, income, employment status, stage, comorbidity, BMI, alcohol drinking, current smoking, MET, treatment type, years from survey date to diagnosis date

### Final multiple proportional hazard regression analyses of QOL adjusted for independent demographic and clinical indicators of survival

**Fig. 1:**
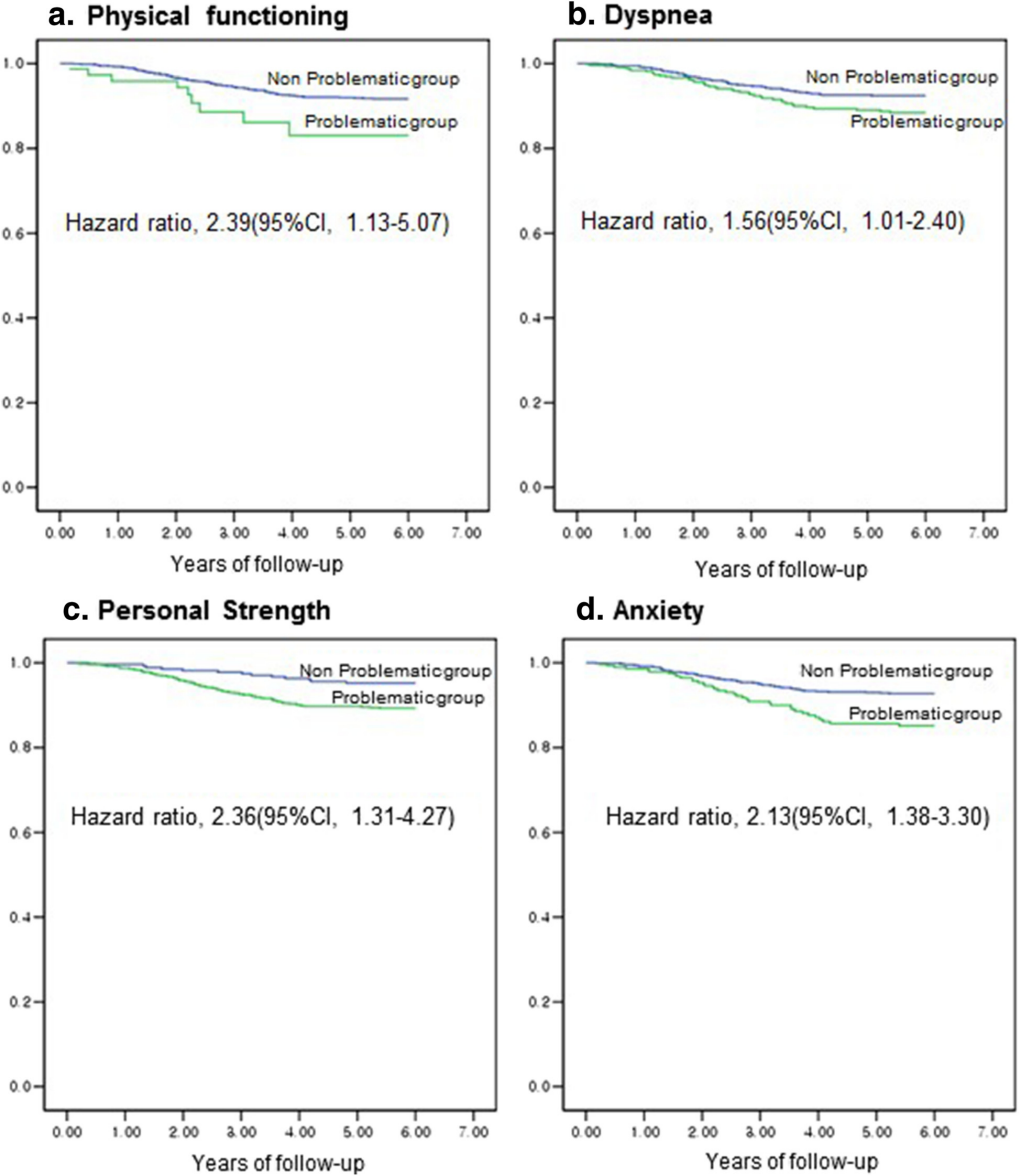
Survival graph by among Lung Cancer Patients. Abbreviations: CI, Confidence Interval; Reference was the non-problematic group. **a** The hazard ratio was multivariable-adjusted for age at diagnosis, sex, cancer stage, BMI after surgery, dyspnea, personal strength, and anxiety. The problematic group for physical functioning was defined as one with a health related quality of life (HRQOL) score of 33or less. **b** The hazard ratio was multivariable-adjusted for age at diagnosis, sex, cancer stage, BMI after surgery, physical functioning, personal strength, and anxiety. The problematic group for dyspnea was defined as one with a symptom scale score of 66 or higher. **c** The hazard ratio was multivariable-adjusted for age at diagnosis, sex, cancer stage, BMI after surgery, physical functioning, dyspnea, and anxiety. The problematic group for personal strength was defined as one with a PTGI factor of 15 or less. **d** The hazard ratio was multivariable-adjusted for age at diagnosis, sex, cancer stage, BMI after surgery, physical functioning, dyspnea, and personal strength. The problematic group for Anxiety was defined as one with a HADS of 8 or higher

After adjustment for independent demographic and clinical indicators of survival, the final multiple proportional hazard regression analyses of QOL showed that physical functioning (aHR 2.39, 95 % CI 1.13–5.07) and dyspnea (aHR 1.56, 95 % CI 1.01–2.40) from the EORTC QLQ-C30, personal strength (aHR 2.36, 95 % CI 1.31–4.27) from the PTGI, and anxiety (aHR 2.13, 95 % CI 1.38–3.30) from the HADS did not lose their independent prognostic power of survival (Table 4 and Fig. 1). In addition, an overweight BMI >23 decreased the hazard of mortality in the final model (aHR for a BMI <23; 1.75, 95 % CI 1.16–2.64; Table 4).

**Table 4:** Multiple proportional hazard regression analyses of QOL adjusted for independent demographic and clinical indicators of survival

	Variable	aHR^a^	95 % CI	
Age (years)	<65	1		
	≥65	1.84	1.19	2.83
Sex	Women	1		
	Men	2.52	1.29	4.93
Stage	stage 0–I	1		
	stage II–III	1.70	1.13	2.56
BMI at the time of survey (kg/m^2^)	≥23	1		
	<23	1.75	1.16	2.64
Physical functioning	>33.33	1		
	≤33.33	2.39	1.13	5.07
Dyspnea	<66.66	1		
	≥66.66	1.56	1.01	2.40
PTGI_Personal strength	≥15	1		
	<15	2.36	1.31	4.27
HADS_Anxiety	<8	1		
	≥8	2.13	1.38	3.30

*Abbreviations*: *aHR* adjusted hazard ratio, *CI* confidence interval, *BMI* body mass index, *PTGI* posttraumatic growth inventory, *HADS* hospital anxiety and depression

^a^Final model of multiple proportional hazard regression analysis including variables identified as independent predictors that showed statistical significance in each of univariate analyses and adjusted proportional hazard regression analyses including QOL

## Discussion

The data obtained from this study suggest that QOL deficits after completion of lung cancer treatment are significantly associated with the prognosis for overall survival (OS). Because clinicians and patients need more information on the patient’s prognosis in terms of the disease-free interval after the completion of cancer treatment when laboratory and imaging techniques cannot provide more prognostic information than the known prognostic factors, a QOL assessment in disease-free lung cancer survivors at the completion of cancer treatment would contribute significantly to predicting patient survival.

After controlling for these covariates, the indication of a clinically-deficient QOL after attaining disease-free status remains clinically meaningful. Therefore, these findings highlight the importance of assessing QOL in survivors after completion of lung cancer treatment.

To the best of our knowledge, this is the first study to report on the prognostic value of QOL scores using a large sample of disease-free survivors with lung cancer after the completion of lung cancer treatment. Our findings are consistent with those of other studies that investigated the importance of pretreatment QOL assessment in patients with non-small cell lung cancer (NSCLC) [12, 33, 34]. Earlier studies suggested that pretreatment scores for global QOL [5, 6], anorexia [4], pain [4–7], and fatigue [4] are the strongest independent prognostic factors, even after the adjustment for established prognostic variables such as age, sex, and stage.

The prognosis of disease-free survivors with NSCLC is significant for both clinical and basic research [20]. The identification of prognostic factors can help provide information for cancer survivors, as well as aid physicians in choosing the best methods for surveillance and intervention. In addition, prognostic QOL factors including survivors’ overall functioning and well-being can be used as supportive monitoring tools in oncology practice [2, 35]. However, no previous studies have addressed the prognostic significance of QOL scores for disease-free lung cancer survivors who are regarded to have a relatively good prognosis after the completion of active cancer treatment.

Symptoms such as anorexia, fatigue, cough, dyspnea, and pain were associated with pro-inflammatory cytokines and vascular endothelial growth factor levels that were significantly related to survival [4]. Although analyses of QOL items adjusted for age, sex, stage, BMI, and PA showed that the scores for the problematic group displaying anorexia, diarrhea, fatigue, cough, and depression were associated with poor survival, they did lose their independent prognostic power of survival in the final multiple proportional hazard regression analyses. Anorexia, diarrhea, and fatigue could be a cause or a consequence of malnutrition, or indicative of subsequent deterioration in the general health status of patients with cancer [36].

Physical functioning, dyspnea, personal strength, and anxiety did not lose their independent prognostic power of survival in the final multiple proportional hazard regression analyses of QOL with independent demographic and clinical indicators of survival. These results could be due to prognostic factors other than QOL scores, and different cut-offs used to dichotomize each scale. Additionally, it is possible that anorexia, diarrhea, fatigue, cough, and depression are strong prognostic variables for survival in advanced lung cancer during clinical trials or after the treatment [24, 33]. Physical functioning, dyspnea, personal strength, and anxiety are likely to have a better prognostic value than anorexia, diarrhea, fatigue, cough, and depression in disease-free lung cancer survivors.

These findings may indicate a disease progression or recurrence that physical examination by a clinician, tumor marker evaluation, and imaging studies (such as computed tomography, magnetic resonance imaging, and positron emission tomography) could not detect [23]. It is also possible that individuals with poor QOL, or those who are not motivated, may be less likely to adhere to their medical treatment plans [12] and good health behaviors (such as moderate-to-vigorous PA) that are independent predictors of mortality in disease-free lung cancer survivors [23, 37, 38]. Therefore, this study suggests the possibility of new prognostic value reflecting long-term survival in disease-free survivors with lung cancer after the completion of active treatment [23]. These findings must be validated prospectively in further studies that should investigate QOL, adherence to cancer treatment, psychoneuroimmunology [13], circulating tumor cells [39], and survival in a different cohort of patients with lung cancer [36].

Additionally, intervention studies will be required to examine the possibility that interventions designed for improving QOL, such as physical functioning, anxiety, and personal strength in patients with lung cancer, may improve survival [12, 40].

Our findings indicate that assessment of QOL should be incorporated into routine oncology clinical practice [21, 24]. This may present certain obstacles, such as an increased burden on the patient [7]. However, if the advantage of such a comprehensive assessment outweighs the disadvantage of its poor applicability in clinical practice [5], repeated assessment of QOL would have significant importance in medical decisions such as the diagnosis of progression and clinical intervention. Our next objective is to develop and test a predictive model for survival in order to identify patients who are experiencing deficits in QOL. The use of a simple questionnaire in clinical practice may benefit patients and provide specific interventions tailored to improve patient QOL and survival [12].

This study has limitations in interpretation and generalization. First, it included only disease-free lung cancer patients from selected hospitals who survived at least 1 year after surgery. Thus, it might not represent the general population of lung cancer survivors. Second, we lacked evaluations of QOL changes over time. QOL values are dynamic, and changes might be associated with long-term survival. Third, this study only addressed overall mortality and did not include cancer-specific mortality and non-cancer mortality. Further studies that include cancer-specific mortality and non-cancer mortality would be helpful for interpreting the prognostic value of QOL in lung cancer. Finally, the participants were surveyed at different time intervals from the time of their diagnosis; we have adjusted for this as a co-variable.

## Conclusion

 This prospective study of a large cohort of survivors with lung cancer suggests that patient-reported QOL outcomes after the completion of active treatment has independent prognostic value for long-term survival. We propose that the assessment of QOL should be incorporated into routine oncology clinical practice.

## Abbreviation

BMI, body mass index; HADS, hospital anxiety and depression scale; HRQOL, health related quality of life; NSCLC, non-small cell lung cancer; OS, overall survival; PA, physical activity; PTGI, posttraumatic growth inventory
